# The Anaerobic Capacity of Cross-Country Skiers: The Effect of Computational Method and Skiing Sub-technique

**DOI:** 10.3389/fspor.2020.00037

**Published:** 2020-04-15

**Authors:** Erik P. Andersson, Dionne A. Noordhof, Nestor Lögdal

**Affiliations:** ^1^Department of Health Sciences, Swedish Winter Sports Research Centre, Mid Sweden University, Östersund, Sweden; ^2^Department of Neuromedicine and Movement Science, Faculty of Medicine and Health Science, Centre for Elite Sports Research, Norwegian University of Science and Technology, Trondheim, Norway

**Keywords:** cross-country skiing, diagonal stride, double poling, gross efficiency, MAOD, maximal accumulated oxygen deficit method, metabolic demand, time trial

## Abstract

Anaerobic capacity is an important performance-determining variable of sprint cross-country skiing. Nevertheless, to date, no study has directly compared the anaerobic capacity, determined using the maximal accumulated oxygen deficit (MAOD) method and gross efficiency (GE) method, while using different skiing sub-techniques.

**Purpose:** To compare the anaerobic capacity assessed using two different MAOD approaches (including and excluding a measured y-intercept) and the GE method during double poling (DP) and diagonal stride (DS) cross-country skiing.

**Methods:** After an initial familiarization trial, 16 well-trained male cross-country skiers performed, in each sub-technique on separate occasions, a submaximal protocol consisting of eight 4-min bouts at intensities between ~47–78% of $\dot{\text{V}}$O_2peak_ followed by a 4-min roller-skiing time trial, with the order of sub-technique being randomized. Linear and polynomial speed-metabolic rate relationships were constructed for both sub-techniques, while using a measured y-intercept (8+*Y*_LIN_ and 8+Y_POL_) or not (8–Y_LIN_ and 8–Y_POL_), to determine the anaerobic capacity using the MAOD method. The average GE (GE_AVG_) of all eight submaximal exercise bouts or the GE of the last submaximal exercise bout (GE_LAST_) were used to calculate the anaerobic capacity using the GE method. Repeated measures ANOVA were used to test differences in anaerobic capacity between methods/approaches.

**Results:** A significant interaction was found between computational method and skiing sub-technique (*P* < 0.001, η^2^ = 0.51) for the anaerobic capacity estimates. The different methodologies resulted in significantly different anaerobic capacity values in DP (*P* < 0.001, η^2^ = 0.74) and in DS (*P* = 0.016, η^2^ = 0.27). The 8-Y_POL_ model resulted in the smallest standard error of the estimate (SEE, 0.24 W·kg^−1^) of the MAOD methods in DP, while the 8-Y_LIN_ resulted in a smaller SEE value than the 8+*Y*_LIN_ model (0.17 vs. 0.33 W·kg^−1^) in DS. The 8-Y_LIN_ and GE_LAST_ resulted in the closest agreement in anaerobic capacity values in DS (typical error 2.1 mL O_2_eq·kg^−1^).

**Conclusions:** It is discouraged to use the same method to estimate the anaerobic capacity in DP and DS sub-techniques. In DP, a polynomial MAOD method (8-Y_POL_) seems to be the preferred method, whereas the 8-Y_LIN_, GE_AVG_, and GE_LAST_ can all be used for DS, but not interchangeable, with GE_LAST_ being the least time-consuming method.

## Introduction

As suggested by the performance model introduced by Joyner and Coyle (2008), performance power output or speed is determined by the total metabolic rate (i.e., the sum of the oxygen uptake [$\dot{\text{V}}{\text{O}}_{2}$] and oxygen [O_2_] deficit) multiplied with the gross mechanical efficiency. Losnegard et al. (2012) were the first to determine the O_2_ deficit or anaerobic capacity, determined as the accumulated O_2_ (ΣO_2_) deficit, during a treadmill roller-skiing sprint time trial. They showed that the relative anaerobic contribution was ~26% and that the between-subject variation in sprint performance was more related to differences in anaerobic capacity than to differences in aerobic capacity. For athletes and coaches, it is likely more appealing to look at how within-subject changes in performance are associated with changes in physiological variables such as the performance $\dot{\text{V}}{\text{O}}_{2}$ and anaerobic capacity. Andersson et al. (2016) showed that the within-subject variance of four successive sprint time trial performances, conducted within ~3 h, could be explained by 69% and 11% of variations in anaerobic metabolic rate (i.e., O_2_ deficit as a rate) and $\dot{\text{V}}{\text{O}}_{2}$, respectively. Moreover, when sprint time trial performance was assessed multiple times during a skiing season it was shown that training resulted in a significant improvement in sprint time trial performance, which was not accompanied by significant changes in peak $\dot{\text{V}}{\text{O}}_{2}$ ($\dot{\text{V}}{\text{O}}_{2\text{peak}}$), but was related to a significant improvement in ΣO_2_ deficit during the season (Losnegard et al., 2013). Altogether, these studies show that anaerobic capacity is an important performance-determining variable for sprint cross-country skiing and that anaerobic capacity should be monitored regularly.

To determine anaerobic capacity during high-intensity exercises, such as a time trial, different methodologies can be used. The most extensively used method to determine anaerobic capacity for supramaximal roller-skiing on a treadmill is the maximal accumulated oxygen deficit (MAOD) method (Losnegard et al., 2012; Andersson and McGawley, 2018; Losnegard, 2019). This method is based on determining a linear relationship between speed (or power output) and submaximal $\dot{\text{V}}{\text{O}}_{2}$ (Medbø et al., 1988). Subsequently, the $\dot{\text{V}}{\text{O}}_{2}$ demand corresponding to supramaximal speeds (or power outputs) can be estimated using extrapolation and the ΣO_2_ deficit can be calculated by subtracting the accumulated VO_2_ uptake from the accumulated *VO*_2_ demand. The MAOD method, as introduced by Medbø et al. (1988), requires the construction of a linear relationship between treadmill speed and submaximal $\dot{\text{V}}{\text{O}}_{2}$ (Medbø and Tabata, 1989), based on a 10 × 10-min discontinuous submaximal protocol performed over several days, which makes this protocol inconvenient from a practical perspective. Therefore, more time-efficient continuous submaximal protocols performed on one day, included as a warm-up before the supramaximal exercise bout, have been used when testing the anaerobic capacity of well-trained and/or elite cross-country skiers (Losnegard et al., 2012; Losnegard and Hallén, 2014; Andersson and McGawley, 2018). Other potential problems with the traditional MAOD method may be related to the issue of linearity between speed and submaximal $\dot{\text{V}}{\text{O}}_{2}$ (or power output) (Bangsbo, 1992, 1996). In classic cross-country roller-skiing, Sandbakk et al. (2016) intended to determine the ΣO_2_ deficit using both diagonal stride and double poling sub-techniques with the MAOD method. However, since the relationship between power output and $\dot{\text{V}}{\text{O}}_{2}$ was non-linear for most of the skiers during double poling, the conventional MAOD method was inappropriate for estimating the ΣO_2_ deficit.

Another method used to determine the anaerobic energy contribution to sprint cross-country skiing is the gross efficiency (GE) method (Andersson et al., 2016, 2017; Andersson and McGawley, 2018) as introduced by Serresse et al. (1988). The GE method requires one submaximal exercise bout at a steady-state intensity (respiratory exchange ratio <1.00) just below the second ventilatory threshold to determine GE and a supramaximal exercise bout. Using the GE method, the anaerobically attributable mechanical work (i.e., the mechanical variant of the anaerobic capacity) can be calculated by subtracting the aerobically attributable mechanical power output (calculated from $\dot{\text{V}}{\text{O}}_{2}$, the energy equivalent for oxygen and GE) from the total mechanical power output and integrate the anaerobically attributable mechanical power output over time (Noordhof et al., 2011). This mechanical variant of the anaerobic capacity can, in addition, be converted and expressed as an O_2_ deficit (Noordhof et al., 2011). When using the GE approach, it is assumed that GE plateaus and remains constant during supramaximal exercise (de Koning et al., 2012). In previous studies on classic cross-country skiing, GE has been observed to be speed independent for diagonal stride but not for double poling (Andersson et al., 2017; Andersson and McGawley, 2018) which makes the traditional GE method more suitable for diagonal stride than double poling (Andersson et al., 2017). An alternative method for estimating the ΣO_2_ deficit during supramaximal exercise is to analyze the fast component of the $\dot{\text{V}}{\text{O}}_{2}$ recovery after exercise (anaerobic alactic source) and the delta increase in blood lactate concentration (anaerobic lactic source; i.e., the peak blood lactate concentration minus the baseline value multiplied with a VO_2_ equivalent of 3 mL·kg^−1^) (di Prampero et al., 1973; di Prampero, 1981; Beneke et al., 2002). This method is favorable for exercise where the movement economy and/or GE cannot be accurately determined (Guidetti et al., 2007), or when sub-maximally determined GE is not considered to reflect the GE during the supramaximal exercise which has been observed for all-out cycle exercise of short duration (Beneke et al., 2002).

To date, there are only two studies that have compared the anaerobic capacity estimated using the MAOD method and GE method (Noordhof et al., 2011; Andersson and McGawley, 2018), one involving cycle ergometry (Noordhof et al., 2011) and one involving diagonal stride roller-skiing exercise (Andersson and McGawley, 2018). Andersson and McGawley (2018) found, in disagreement with Noordhof et al. (2011), a significant difference in accumulated oxygen demand and anaerobic capacity between methods. However, also Noordhof et al. (2011) showed that individual differences in anaerobic capacity between methods existed, and therefore suggested not to use these methods interchangeably. Of note, Noordhof et al. (2011) used a discontinuous protocol of 10 × 10-min submaximal cycling bouts evenly distributed between 30 and 90% of $\dot{\text{V}}{\text{O}}_{2\text{max}}$ performed on 2 days to determine the linear relationship between power output and $\dot{\text{V}}{\text{O}}_{2}$, with GE based on a single submaximal stage. This differs from the continuous protocol employed by Andersson and McGawley (2018) for roller-skiing, where 4 × 4-min submaximal exercise intensity stages evenly distributed between 60 and 82% of $\dot{\text{V}}{\text{O}}_{2\text{max}}$ were used, and GE was determined as an average value based on the same exercise intensities. These different testing protocols and exercise modes may be explanatory factors for the divergent findings.

In comparison to other cyclic sports, one unique aspect of cross-country skiing is the different sub-techniques involved in the classic and ski-skating styles, whereby the choice of sub-technique is both speed and incline dependent (Nilsson et al., 2004; Kvamme et al., 2005; Andersson et al., 2010, 2017). Nevertheless, to date, no study has directly compared the anaerobic capacity, determined using the MAOD, GE method and/or alternative methods, between different sub-techniques. Therefore, this study aimed to compare anaerobic capacity estimates by using two linear and two polynomial MAOD approaches (including and excluding a measured y-intercept in the respective regressions) and two GE approaches for the double poling (DP) and diagonal stride (DS) sub-techniques in elite cross-country skiers. In comparison to the protocol used by Andersson and McGawley (2018), this study was designed to include: (I) more submaximal exercise stages within a slightly wider exercise intensity range, (II) measure baseline $\dot{\text{V}}{\text{O}}_{2}$ (y-intercept of the relationship between speed and aerobic metabolic rate), and (III) determine the anaerobic capacity for both the DP and DS sub-techniques.

## Methods

### Participants

Sixteen well-trained male cross country skiers (26 ± 5 years, 182 ± 6 cm, 77.3 ± 6.7 kg), competing at the national level were recruited for this study, which was preapproved by the Regional Ethical Review Board of Umeå University, Umeå, Sweden (#2018-154-31 M). A maximal oxygen uptake of at least 60 ml·kg^−1^·min^−1^ was set as an inclusion criterion. Participants were instructed to engage only in low-intensity exercise the day before testing and consume carbohydrate-rich meals, to ensure adequate muscle glycogen content. Participants received both written and verbal information about the experimental protocol and potential risks involved before they provided written informed consent.

### Study Overview

**Figure 1:**
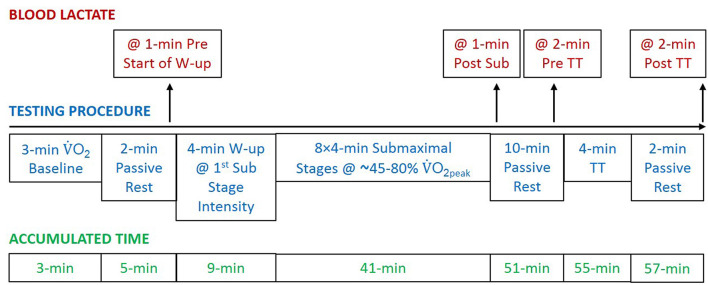
A schematic overview of the protocol used for both double poling (at a 1.5° treadmill incline) and diagonal stride (at a 6.5° treadmill incline). After a short rest, the subjects were fitted with the equipment for cardiopulmonary measurements at rest (baseline oxygen uptake [$\dot{\text{V}}{\text{O}}_{2}$]). Capillary blood samples for the determination of blood lactate concentration were collected four times. Abbreviations: @, at; W-up, warm-up; Sub, submaximal; TT, time trial; $\dot{\text{V}}$O_2peak_, peak oxygen uptake.

Participants completed a familiarization session on the treadmill before their first test day to minimize the effect of learning on time trial performance (Foster et al., 2009). On separate test days, participants completed in each sub-technique (DP and DS) a continuous submaximal protocol consisting of eight 4-min stages at intensities between ~47–78%, and a 4-min supramaximal roller-skiing time trial. A schematic overview of the test protocol is illustrated in Figure 1. The two test days were completed within a 2-week period, separated by at least 2 days, and the order of sub-technique was randomized. The submaximal protocols for DP and DS were intensity-matched based on average GE values from previous studies (Andersson et al., 2017; Andersson and McGawley, 2018). The metabolic demand was estimated by dividing power output for each submaximal speed with GE, and set relative to the skier's previously measured peak/maximum $\dot{\text{V}}{\text{O}}_{2}$ in DS and assuming a peak/maximum $\dot{\text{V}}{\text{O}}_{2}$ in DP that is 4% lower than DS based on previous data by Andersson et al. (2017).

### Equipment and Measurements

All tests were performed on a treadmill specifically designed for roller-skiing (Rodby Innovation AB, Vänge, Sweden) that allows the athlete to freely adjust the speed by moving forward or backward on the treadmill, which makes time-trial tests possible (Swarén et al., 2013). Distance completed during the time trial was automatically logged at a rate of 2.46 Hz and linearly interpolated to second-by-second data. Participants completed all testing using the same pair of classic roller skis (Pro-Ski C2, Sterners, Dala-Järna, Sweden) in order to minimize potential variations in rolling resistance. The coefficient of rolling resistance (μR) of the skis was on average 0.0215 and determined as previously described (Ainegren et al., 2008). In order to avoid changes in rolling resistance during test-sessions the skis were pre-warmed in a heat box for a minimum of 60 min before testing and kept in the heat box whilst not used. Participants used their own poles, which were fitted with rubber tips specially designed for treadmill skiing. Respiratory measurements were performed using an AMIS 2001, model C (Innovision AS, Odense Denmark). The gas analyzers were calibrated with a known reference gas (16.0 O_2_ and 4.5% CO_2_, Air Liquide, Kungsängen, Sweden) and the flowmeter was calibrated with a 3-L syringe at low, medium and high flow rates (Hans Rudolph, Kansas City, Missouri, USA) before the start of each test. Ambient temperature was 19.5 ± 0.5°C at a relative humidity of 21 ± 6% and both were monitored with a Vaisala PTU200 (Vaisala Oy, Helsinki, Finland). Heart rate was monitored using a chest strap and wristwatch (V800, Polar Electro Oy, Kempele, Finland). Blood lactate concentration was determined using a Biosen S_Line (EKF diagnostics, Magdeburg, Germany) calibrated with a known standard solution of 12 mmol·L^−1^.

### Testing Procedures

Upon arrival to the laboratory body mass of the participants, with and without equipment was measured using an electronic scale (Seca 764, Hamburg, Germany) after which participants rested in a supine position. Before the start of the submaximal exercise protocol, a 3-min baseline $\dot{\text{V}}{\text{O}}_{2}$ measurement was collected while the participant was standing still on the treadmill that was preceded by ~5 min of supine rest and ~5 min of seated rest. The DP protocol was performed at an incline of 1.5° and the DS protocol was performed at an incline of 6.5°. Depending on the performance level of the athlete (based on previous maximum/peak $\dot{\text{V}}{\text{O}}_{2}$ test results for DS), the starting speed was either 6 or 6.5 km·h^−1^ for the DS protocol and either 12.6 or 13.8 km·h^−1^ for the DP protocol. The speed was increased by 0.5 and 1.2 km·h^−1^ for DS and DP, respectively, up to a final speed of either 9.5 or 10 km·h^−1^ for DS and 21 or 22.2 km·h^−1^ for DP. Both protocols consisted of 8 × 4-min submaximal stages (with the exception of the first stage that lasted 8 min), followed by a 10-min passive rest and a 4-min time trial at a self-selected speed.

The only instruction participants received before the time trial was to cover as much distance as possible. Capillary blood samples (20 μL) were taken from a fingertip for the assessment of the blood lactate concentration 1 min before the submaximal exercise protocol, 1 min after cessation of the last submaximal stage, 2 min prior to, and 2 min after the time trial. The skiers rated their perceived exertion (RPE) after the last submaximal stage as well as immediately after the time trial using the 10-point scale of Foster et al. (2001). Participants received feedback on elapsed time every 30 s but no feedback regarding their speed during the time trial. Respiratory and heart rate data were collected continuously during the submaximal protocol and time trial. The highest 30-s moving average during the time trial was used to calculate $\dot{\text{V}}$O_2peak_ and peak ventilation rate, while peak heart rate was obtained as the highest 1-s value. Peak respiratory exchange ratio (RER) was taken over the same period as the $\dot{\text{V}}$O_2peak_. During all testing, participants were secured with a safety harness suspended from the ceiling and connected to an emergency brake, which immediately stopped the treadmill in case of a fall.

### Calculations

#### Submaximal Roller-Skiing

The power output for submaximal roller-skiing was calculated as the sum of the power exerted to overcome the rolling resistance and to elevate body mass and skiing equipment (m_sys_) against gravity:

(1) $$\begin{array}{l} Power\;output\;\left[W\right]=\;vm_{sys}\left(g\;\text{sin}\left(\alpha \right)+u\mu_{R}g\;\text{cos}\left(\alpha \right)\right) \end{array}$$

where *g* is gravitational acceleration, *v* is the treadmill speed [m·s^−1^], μ_R_ is the rolling resistance coefficient and α is the treadmill incline (Andersson and McGawley, 2018). The m_sys_ was 80.7 ± 6.8 kg. Energy expenditure was calculated from $\dot{\text{V}}{\text{O}}_{2}$ (L·min^−1^) and RER ($\dot{\text{V}}$${\text{CO}}_{2}\cdot \dot{\text{V}}{\text{o}}_{2}^{-1}$) according to the equation introduced by Weir (1949) and then converted into a metabolic rate. Metabolic rate was based on the average $\dot{\text{V}}{\text{O}}_{2}$ and RER values (≤1.00) during the final minute of each stage of the submaximal exercise protocol.

(2) $$\begin{array}{l} Metabolic\;rate\;[W]=\;\frac{4184\dot{(V}O_{2}\left(1.1RER+3.9\right))}{60} \end{array}$$

Energy cost relative to m_sys_ was expressed as:

(3) $$\begin{array}{l} Energy\;cost\;\left[{J.kg}^{-1}{.m}^{-1}\right]=\;\frac{Metabolic\;rate}{vm_{sys}} \end{array}$$

GE was calculated using the following equation:

(4) $$\begin{array}{l} GE=\;\frac{Power\;output}{Metabolic\;rate} \end{array}$$

Net efficiency was calculated as:

(5) $$\begin{array}{l} Net\;efficiency=\;\frac{Power\;output}{Metabolic\;rate-MR_{BL}} \end{array}$$

where MR_BL_ is the baseline metabolic rate calculated from a 3-min baseline $\dot{\text{V}}{\text{O}}_{2}$ and RER measurement with the participant standing still on the treadmill (measured prior to the warm-up). Delta efficiency was calculated by dividing the delta increase in power output by the delta increase in metabolic rate based on the linear regression between metabolic rate and power output over the eight submaximal exercise intensities (i.e., the reciprocal value of the slope of the regression equation). Neither net efficiency nor delta efficiency were used for estimating the anaerobic capacity.

#### Estimating the Anaerobic Capacity Using the Linear and Polynomial MAOD Methods

Since the traditional MAOD method for estimating a supramaximal $\dot{\text{V}}{\text{O}}_{2}$ demand is based on a linear relationship between speed or power output and submaximal $\dot{\text{V}}{\text{O}}_{2}$, changes in substrate utilization are not considered. Thus, a speed or power output vs. metabolic rate relationship should be more appropriate due to the different energetic equivalents of fat and carbohydrate oxidation (Andersson and McGawley, 2018). Therefore, a linear relationship between treadmill power output and metabolic rate during the final minute of each of the 8 × 4-min submaximal stages was derived for each participant with the baseline metabolic rate as a Y-intercept (i.e., metabolic rate at zero speed) included in (8+*Y*_LIN_) or excluded from (8-Y_LIN_) the model. In the latter case, the Y-intercept was based on all data points in the regression (i.e., not forced). Second-degree polynomial relationships (8+Y_POL_ and 8-Y_POL_) were also derived based on the same data points. The four regression equations (two linear and two polynomial) were used to estimate the required instantaneous metabolic rate during the 4-min time trial (MR_TT_req_) at each 1-s time-point for DP (1.5°) and DS (6.5°), respectively. The power output during the time trial was calculated according to Eq. 1.

The instantaneous anaerobic metabolic rate (MR_an_) at each 1-s time-point (*t*) of the time trial was expressed as:

(6) $$\begin{array}{l} MR_{an,t}\;\left[{J\cdot \;s}^{-1}\right]=\;MR_{TT\text{\_}req,t}-\;MR_{ae,t} \end{array}$$

where MR_ae_ is the aerobic metabolic rate calculated as described in Eq. 2.

The total anaerobic energy production (E_an_ [J]) was calculated by integrating MR_an_ over the 4-min time trial. The anaerobic energy production was, in addition, converted to an ΣO_2_ deficit by multiplying the E_an_ with a constant of 0.047801 (mL O_2_ equivalent per joule) according to Weir (1949) and assuming 100% carbohydrate utilization during the supramaximal time trial. $\dot{\text{V}}$O_2peak_ (L·min^−1^) during the time trial was, in addition, converted to a peak aerobic metabolic rate by using Eq. 2 and assuming a 100% carbohydrate utilization (i.e., using an RER of 1.00).

#### Estimating the Anaerobic Capacity Using the GE Methods

To calculate the MR_an_, the submaximal GE calculated as an average GE of all the submaximal stages (GE_AVG_) or the last submaximal stage (GE_LAST_) was used. Here, the MR_TT_req_ at each 1-s time-point of the time trial was calculated by dividing the instantaneous power output using a fixed GE value (i.e., GE_AVG_ or GE_LAST_) where the MR_an_ was given by subtracting the MR_ae_ from the MR_TT_req_ (Eq. 6). To obtain an anaerobic capacity value (i.e., E_an_ [J]), MR_an_ was integrated over time and also expressed as an ΣO_2_ deficit, similarly as for the linear and polynomial MAOD methods.

#### Comparing the Measured GE With Gross Efficiency Derived From the Four Regression Equations (GE_REG_)

The GE based on each of the four regression equations (i.e., GE_REG_) was calculated for each of the submaximal stages as power output divided by metabolic rate, with metabolic rate calculated from the regression equation. This was done to enable a comparison of the measured GE with the GE_REG_ as based on the four MAOD methods. To be able to compare the average supramaximal GE_REG_ during the time trial as based on the different regression equations vs. the GE_AVG_ and GE_LAST_ values, the following calculations were performed. Firstly, the estimated instantaneous GE at each 1-s time-point (*t*) of the 4-min time trial was calculated for 8+*Y*_LIN_, 8-Y_LIN_, 8+Y_POL_ and 8-Y_POL_ as the ratio between power output (calculated similarly as in Eq. 1) and the MR_TT_req_ derived from the linear and polynomial regression equations. Secondly, the estimated instantaneous GE during the time trial was expressed as an average value for each of the four respective methods.

### Statistics

The Statistical Package for the Social Sciences (SPSS 21, IBM Corp., Armonk, NY, USA) was used to carry out statistical analyses and the level of significance was set at α ≤ 0.05. Data were checked for normality by visual inspection of Q-Q plots and histograms together with the Shapiro-Wilks analysis and are presented as mean ± standard deviation (SD), except in the case of RPE, where data are presented as median and interquartile range (IQR). In addition, the different anaerobic capacity estimates were presented as mean and 95% confidence interval. One-way repeated measures ANOVA tests were used to compare GE, net efficiency (NE) and energy cost (EC) between the eight submaximal stages as well as to analyze the regression coefficients based on the submaximal relationships between relative metabolic rate and speed as well as the estimated GE, metabolic requirements and anaerobic capacities during the time trial as determined from the six methods (i.e., 8+*Y*_LIN_, 8-Y_LIN_, 8+*Y*_POL_, 8-Y_POL_, GE_AVG_, and GE_LAST_). A two-way repeated measure ANOVA (6 × 2) was used for the comparison of the six anaerobic capacity estimates between the two sub-techniques and for analyzing the interaction effect. The assumption of sphericity was tested using Mauchly's test and for violated sphericity the degrees of freedom were corrected using the Greenhouse-Geisser correction (i.e., epsilon ≤ 0.75). Eta squared effect size (η^2^) was also reported for the ANOVA tests. Bonferroni α corrections were applied to all ANOVA tests.

The mean difference ± 95% limits of agreement were evaluated for the comparison of the methods by using Bland-Altman calculations (Bland and Altman, 1999). The mean difference was tested with a one-sample *t*-test using a reference value of zero. Relationships between variables were assessed using linear and polynomial (second-degree) regression analyses. The accuracy of the regression equations was assessed with the standard error of the estimate (SEE). The individual delta efficiencies (i.e., 8+*Y*_LIN_ and 8-Y_LIN_) and differences in physiological responses between sub-techniques during the time trials were analyzed with paired *t*-tests, while a Wilcoxon signed-rank test was used for analyzing peak heart rate values. For the one-sample and paired *t*-tests, the standardized mean difference (Hedges' *g*_*av*_, effect size [ES_*Hg*_*av*_]) was computed according to the equations presented by Cumming (2012). In addition, the absolute typical error for the comparisons was computed by taking the SD for the pair-wise mean differences divided by the square root of two. The root mean square error was used to evaluate the discrepancy between GE calculated from the four regression equations and measured GE during the eight stages of submaximal roller-skiing and were compared with a one-way repeated measures ANOVA. The within-athlete coefficient of variation in GE during the eight stages of submaximal roller-skiing was calculated as the within-athlete SD divided by the within-athlete mean.

## Results

### Submaximal Data

**Figure 2:**
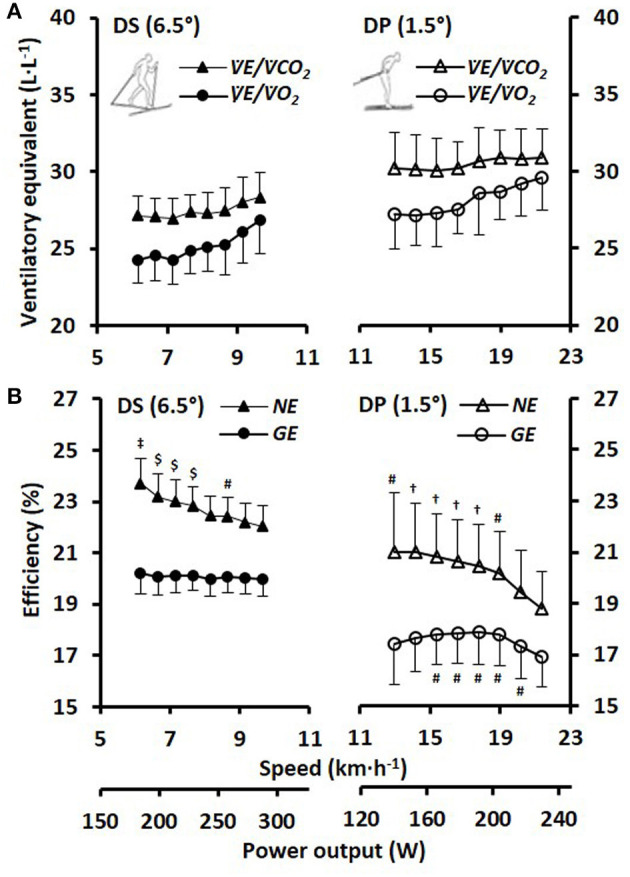
**(A)** Ventilatory equivalents of oxygen ($\dot{\text{VE}}\cdot \dot{\text{V}}{\text{O}}_{2}^{-1}$) and carbon dioxide ($\dot{\text{VE}}\cdot \dot{\text{V}}{\text{CO}}_{2}^{-1}$) and **(B)** net efficiency (NE) and gross efficiency (GE) for the respective sub-techniques plotted against skiing speed and the average power output for the eight 4-min stages of submaximal treadmill roller-skiing (SUB_1−8_) with diagonal stride (DS) and double poling (DP) at inclines of 6.5 and 1.5°, respectively. The values are presented as mean ± SD. Statistical comparisons were performed for GE and NE.^‡^Statistically significantly different (SSD) from SUB_4−8_. ^$^SSD from SUB_5−8_. ^†^SSD from SUB_7−8_. ^#^ SSD from SUB_8_.

**Figure 3:**
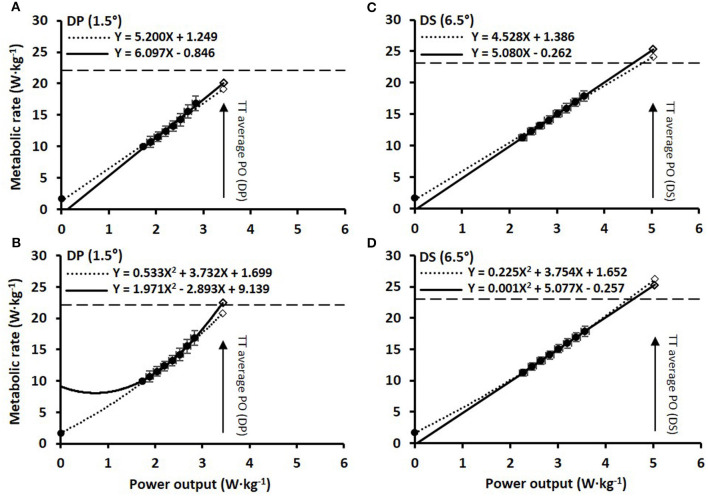
The various regression models between mean ± SD power output and metabolic rate relative to system mass of the skier during 8 × 4-min stages of continuous submaximal roller-skiing together with the estimated total metabolic requirements (open tilted squares) at the average power output (PO) attained during the 4-min time-trial (TT). **(A)** linear relationship for double poling (DP) at 1.5° using a Y-intercept (8+Y_LIN_), dashed line, and excluding a Y-intercept value, solid line (8–Y_LIN_); **(B)** the same data for DP as in **(A)** but using polynomial regressions; **(C,D)** diagonal stride (DS) roller-skiing at 6.5° using the same regression models as in **(A,B)**. The dashed horizontal lines indicate the peak aerobic metabolic rate during the respective TTs.

**Figure 4:**
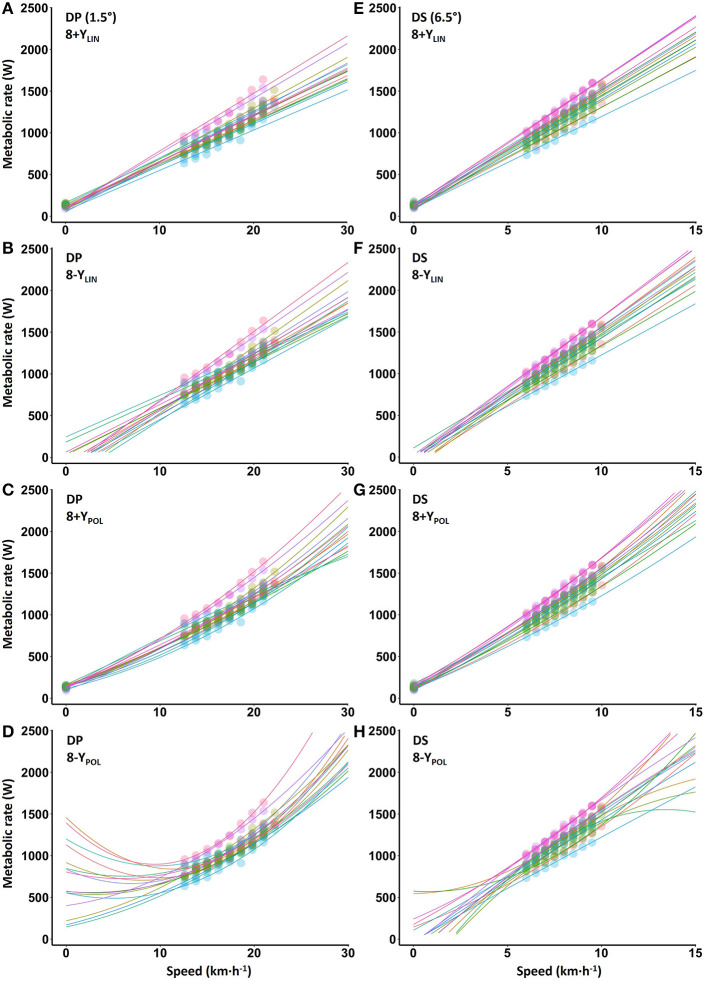
Individual regressions for submaximal metabolic rate plotted against treadmill roller-skiing speed using the double poling (DP) at 1.5° and diagonal stride (DS) at 6.5°. 8+*Y*_LIN_ **(A,E)** and 8-Y_LIN_ **(B,F)**, the 8 × 4-min linear regressions with the baseline metabolic rate as a Y-intercept either included (8+Y) or excluded (8–Y). 8+Y_POL_ **(C,G)** and 8–Y_POL_ **(D,H)**, the 8 × 4-min polynomial (second degree) regressions with the baseline metabolic rate as a Y-intercept either included (8+Y) or excluded (8–Y).

**Figure 5:**
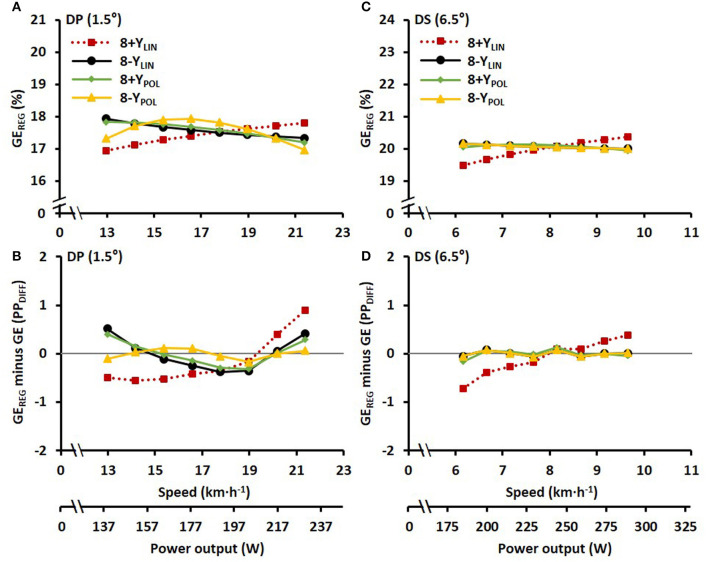
**(A,C)** Average gross efficiency calculated from the four regression equations (GE_REG_) based on submaximal power output and metabolic rate for the double poling (DP) and diagonal stride (DS) sub-techniques plotted against treadmill roller-skiing speed and the average power output. **(B,D)** The average percentage point difference (PP_DIFF_) between GE_REG_ and the measured gross efficiency (GE) for the same sub-techniques and speeds. Where 8+Y_LIN_ and 8–Y_LIN_ are the 8 × 4-min linear regressions with the baseline metabolic rate as a Y-intercept either included (8+Y) or excluded (8–Y), while 8+Y_POL_ and 8–Y_POL_ are second-degree polynomial relationships based on the same data points. The gray horizontal line represents the identity line between GE_REG_ and GE.

The blood lactate concentrations 1-min prior to and 1-min after the submaximal roller-skiing were in DP 1.7 ± 0.5 and 3.5 ± 1.2 mmol·L^−1^, respectively, and in DS 1.5 ± 0.4 and 2.7 ± 1.1 mmol·L^−1^, respectively. The cardiorespiratory variables, two various concepts of efficiency (i.e., net efficiency and GE), together with relative energy cost at each of the eight submaximal speeds for DP and DS, are shown in Table 1 and Figure 2. In DP, GE and net efficiency were both dependent on speed (GE: F_2, 31_ = 6.45, *P* = 0.004, η^2^ = 0.30; NE: F_2, 29_ = 19.37, *P* < 0.001, η^2^ = 0.56), while in DS only net efficiency was dependent on speed (GE: F_7, 105_ = 1.32, *P* = 0.247, η^2^ = 0.08; NE: F_7, 105_ = 38.80, *P* < 0.001, η^2^ = 0.72) (Figure 2). The within-athlete coefficient of variation in GE for the eight submaximal stages was 3.4 ± 1.0 and 1.3 ± 0.5% for DP and DS, respectively. The delta efficiency for the 8+*Y*_LIN_ and 8-Y_LIN_ regressions was for DP 19.3 ± 1.5 and 16.7 ± 2.5%, respectively (*P* < 0.001, ES_*Hg_av*_ = 1.2) and was for DS, 22.1 ± 0.7 and 19.7 ± 1.1%, respectively (*P* < 0.001, ES_*Hg_av*_ = 2.4). The mean ± SD power output and metabolic rate during the eight stages, together with the regression lines are displayed for DP in Figure 3A for the 8+*Y*_LIN_ and 8-Y_LIN_ models and Figure 3B for the 8+Y_POL_ and 8-Y_POL_ models with the same variables presented for DS in Figures 3C,D. All individual regression lines between speed and metabolic rate of the four different regression models used to estimate the total metabolic requirement during the time trials in DP and DS are shown in Figure 4. The GE for the submaximal stages calculated from the four regression equations (i.e., GE_REG_) and the percentage point differences between GE_REG_ and the measured GE for the four regression methods are shown in Figure 5. The root mean square errors for the percentage point differences between GE_REG_ and measured GE during the eight submaximal stages of roller-skiing are shown in Table 2.

**Table 1:** Mean ± SD of speeds, heart rates, cardiorespiratory variables, and relative energy costs associated with the eight submaximal stages (SUB_1−8_) of double poling and diagonal stride roller-skiing.

	**SUB_1_**	**SUB_2_**	**SUB_3_**	**SUB_4_**	**SUB_5_**	**SUB_6_**	**SUB_7_**	**SUB_8_**
**Double poling (1.5**^**°**^)								
Speed (km·h^−1^)	13.0 ± 0.6	14.2 ± 0.6	15.4 ± 0.6	16.6 ± 0.6	17.8 ± 0.6	19.0 ± 0.6	20.2 ± 0.6	21.4 ± 0.6
Heart rate (% of max)	66 ± 4	70 ± 4	74 ± 5	78 ± 5	81 ± 4	85 ± 4	89 ± 4	92 ± 3
VO2 (mL·kg^−1^[BM]·min^−1^)	30.5 ± 2.7	32.8 ± 2.4	35.2 ± 2.6	37.8 ± 2.5	40.4 ± 2.9	43.3 ± 3.3	47.1 ± 3.6	51.0 ± 3.7
VO_2_ (% of VO_2peak_)	45 ± 3	49 ± 3	52 ± 3	56 ± 4	60 ± 4	65 ± 5	71 ± 5	77 ± 5
Ventilation rate (L·min^−1^)	63.9 ± 9.1	68.5 ± 8.0	74.0 ± 7.8	80.3 ± 8.4	88.8 ± 11.5	95.8 ± 11.7	105.9 ± 12.0	116.5 ± 14.1
Respiratory exchange ratio	0.90 ± 0.04	0.90 ± 0.04	0.91 ± 0.04	0.91 ± 0.04	0.93 ± 0.04	0.93 ± 0.04	0.94 ± 0.04	0.95 ± 0.03
Energy cost (J·kg^−1^[SM]·m^−1^)	2.76 ± 0.24	2.72 ± 0.20	2.70 ± 0.18^#^	2.69 ± 0.17^#^	2.69 ± 0.19^#^	2.70 ± 0.19^#^	2.77 ± 0.20^#^	2.84 ± 0.20
**Diagonal stride (6.5**^**°**^)								
Speed (km·h^−1^)	6.2 ± 0.2	6.7 ± 0.2	7.2 ± 0.2	7.7 ± 0.2	8.2 ± 0.2	8.7 ± 0.2	9.2 ± 0.2	9.7 ± 0.2
Heart rate (% of max)	68 ± 3	73 ± 3	76 ± 3	80 ± 3	84 ± 3	87 ± 3	90 ± 3	93 ± 3
VO2 (mL·kg^−1^[BM]·min^−1^)	34.6 ± 1.6	37.6 ± 1.7	40.4 ± 1.7	43.0 ± 1.9	46.1 ± 2.0	48.7 ± 2.3	51.6 ± 2.1	54.3 ± 2.6
VO_2_ (% of VO_2peak_)	49 ± 2	53 ± 3	57 ± 3	61 ± 3	66 ± 3	69 ± 3	74 ± 4	78 ± 4
Ventilation rate (L$\cdot \overset{-1}{\min}$)	64.7 ± 5.9	71.2 ± 7.3	75.8 ± 8.5	82.6 ± 8.6	89.3 ± 8.8	94.8 ± 9.7	103.7 ± 10.9	112.4 ± 12.3
Respiratory exchange ratio	0.89 ± 0.04	0.91 ± 0.04	0.90 ± 0.03	0.91 ± 0.04	0.92 ± 0.03	0.92 ± 0.04	0.93 ± 0.04	0.94 ± 0.04
Energy cost (J·kg^−1^[SM]·m^−1^)	6.60 ± 0.26	6.65 ± 0.23	6.63 ± 0.22	6.62 ± 0.20	6.67 ± 0.22	6.63 ± 0.22	6.66 ± 0.20	6.67 ± 0.23

*$\dot{V}O_{2}$, oxygen uptake; $\dot{V}O_{2peak}$, peak oxygen uptake; BM, body mass; SM, system mass. Statistical comparisons were performed for energy cost*.

# *statistically significantly different (P < 0.05) from SUB_8_*.

**Table 2:** Mean ± SD of the coefficient of determination (*r*^2^), standard error of estimate (SEE), and the regression coefficients for the six different methods of estimating the metabolic demands and accumulated oxygen (ΣO_2_) deficits during the 4-min time-trial (TT) in double poling and diagonal stride.

	**Method of calculation**							
	**8+Y_LIN_**	**8–Y_LIN_**	**8+Y_POL_**	**8–Y_POL_**	**GE_AVG_**	**GE_LAST_**	***F*-value**	**η^2^**
**Double poling (1.5°)**								
*r*^2^	0.99 ± 0.01^c^	0.98 ± 0.01^c^^,^^d^	1.00 ± 0.00	0.99 ± 0.01	–	–	*F*_(2, 28) =_ 20^$^	0.57
SEE (W·kg^−1^)	0.56 ± 0.18^b^^,^^c^^,^^d^	0.36 ± 0.11^c^^,^^d^	0.32 ± 0.10	0.24 ± 0.11	–	–	*F*_(2, 26) =_ 31^$^	0.68
Y-intercept (W·kg^−1^)	1.25 ± 0.35^b^^,^^c^^,^^d^	−0.86 ± 1.82^c^^,^^d^	1.70 ± 0.21^d^	9.14 ± 4.99	–	–	*F*_(1, 18) =_ 49^$^	0.77
L_coeFf_ (W·kg^−1^*perkm*·*h*^−1^)	0.69 ± 0.05^b^^,^^c^^,^^d^	0.81 ± 0.12^c^^,^^d^	0.50 ± 0.12^d^	−0.38 ± 0.53	–	–	*F*_(1, 18) =_ 57^$^	0.79
Q_coeff_ (W·kg^−1^*perkm*·h^−2^)	–	–	0.01 ± 0.01^d^	0.03 ± 0.02	–	–	–	–
RMSE (GE_REG_ vs. GE [PP])	0.6 ± 0.2^b^^,^^c^^,^^d^	0.4 ± 0.1^c^^,^^d^	0.4 ± 0.1^d^	0.2 ± 0.1	–	–	*F*_(3, 45) =_ 31^$^	0.67
GE_TT_avg_ (%)	18.0 ± 1.2^c^^,^^d^^,^^e^^,^^f^	17.5 ± 1.5^d^^,^^f^	16.6 ± 1.3^d^	15.2 ± 1.4^e^^,^^f^	17.6 ± 1.2^f^	16.9 ± 1.2	*F*_(3, 40) =_ 46^$^	0.75
MR_TT_req_ (W·kg^−1^)	19 ± 1^b^^,^^c^^,^^d^^,^^e^^,^^f^	20 ± 1^c^^,^^d^	21 ± 2^d^^,^^e^	23 ± 3	20 ± 1	20 ± 1	*F*_(2, 27) =_ 42^$^	0.74
MR_TT_req_ (% of MR_ae_peak_)	87 ± 4^b^^,^^c^^,^^d^^,^^e^^,^^f^	91 ± 5^c^^,^^d^	94 ± 7^d^^,^^e^	103 ± 9	89 ± 4	92 ± 5	*F*_(2, 28) =_ 45^$^	0.75
Anaerobic capacity (kJ·kg^−1^)	−0.10 ± 0.20^b^^,^^c^^,^^d^^,^^e^^,^^f^	0.14 ± 0.27^,^^d^	0.33 ± 0.36^d^^,^^e^	0.83 ± 0.48^e^^,^^f^	0.03 ± 0.19^f^	0.22 ± 0.21	*F*_(2, 28) =_ 42^$^	0.74
ΣO_2_ deficit (mL·kg^−1^)	−5 ± 9^b^^,^^c^^,^^d^^,^^e^^,^^f^	6 ± 12^c^^,^^d^	15 ± 16^d^^,^^e^	38 ± 22^e^^,^^f^	1 ± 9^f^	10 ± 10	*F*_(2, 28) =_ 42^$^	0.74
**Diagonal stride (6.5°)**								
*r*^2^	1.00 ± 0.00^c^	0.99 ± 0.00^c^	1.00 ± 0.00^d^	1.00 ± 0.00	–	–	*F*_(3, 45) =_ 14^$^	0.48
SEE (W·kg^−1^)	0.33 ± 0.08^b^^,^^c^^,^^d^	0.17 ± 0.05	0.17 ± 0.05	0.16 ± 0.05	–	–	*F*_(1, 19) =_ 42^$^	0.74
Y-intercept (W·kg^−1^)	1.39 ± 0.22^b^^,^^c^	−0.28 ± 0.75^c^	1.65 ± 0.23	−0.21 ± 4.06	–	–	*F*_(1, 16) =_ 4^*^	0.20
L_coeff_ (W·kg^−1^*perkm*·h^−1^)	1.67 ± 0.05^b^^,^^c^	1.88 ± 0.10^c^	1.39 ± 0.12	1.87 ± 1.08	–	–	*F*_(3, 45) =_ 3^*^	0.16
Q_coeff_ (W·kg^−1^*perkm*·h^−2^)	–	–	0.03 ± 0.01	0.00 ± 0.07	–	–	–	–
RMSE (GE_REG_ vs GE [PP])	0.4 ± 0.1^b^^,^^c^^,^^d^	0.2 ± 0.1	0.2 ± 0.1	0.2 ± 0.1	–	–	*F*_(1, 20) =_ 55^$^	0.79
GE_TT_avg_ (%)	20.8 ± 0.6^b^^,^^c^^,^^e^^,^^f^	19.9 ± 0.7^c^	19.2 ± 0.9^e^^,^^f^	20.2 ± 2.8	20.1 ± 0.6	20.0 ± 0.7	*F*_(1, 16) =_ 4^*^	0.22
MR_TT_req_ (W·kg^−1^)	24 ± 1^b^^,^^c^^,^^e^^,^^f^	25 ± 1^c^	26 ± 2^e^^,^^f^	25 ± 4	25 ± 1	25 ± 1	*F*_(1, 17) =_ 6^*^	0.27
MR_TT_req_ (% of MR_ae_peak_)	105 ± 4^b^^,^^c^^,^^e^^,^^f^	110 ± 4^c^	114 ± 5^e^^,^^f^	110 ± 14	109 ± 4	109 ± 4	*F*_(1, 17) =_ 5^*^	0.26
Anaerobic capacity (kJ·kg^−1^)	0.89 ± 0.17^b^^,^^c^^,^^e^^,^^f^	1.17 ± 0.18^c^	1.41 ± 0.26^e^^,^^f^	1.19 ± 0.76	1.12 ± 0.18	1.15 ± 0.19	*F*_(1, 17) =_ 6^*^	0.27
ΣO_2_ deficit (mL·kg^−1^)	41 ± 8^b^^,^^c^^,^^e^^,^^f^	54 ± 8^c^	65 ± 12^e^^,^^f^	55 ± 35	51 ± 8	53 ± 9	*F*_(1, 17) =_ 6^*^	0.27

*8+Y_LIN_ and 8-Y_LIN_, the 8 × 4-min linear maximal accumulated O_2_ deficit methods with the baseline $\dot{V}O_{2}$ as a Y-intercept either included (8+Y) or excluded (8-Y); 8+Y_POL_ and 8-Y_POL_, the 8 × 4-min polynomial maximal accumulated O_2_ deficit methods with the baseline $\dot{V}O_{2}$ as a Y-intercept either included (8+Y) or excluded (8-Y); GE_AVG_, the gross efficiency method based on the average of eight submaximal stages; GE_LAST_, the gross efficiency method based on the last submaximal stage; L_coeff_, linear coefficient; Q_coeff_, quadratic coefficient; RMSE, the root mean square error for the discrepancy between gross efficiency calculated from the regression equation (GE_REG_) and measured gross efficiency (GE) during the eight stages of submaximal roller-skiing expressed as a percentage point (PP) error; GE_TTavg_, average GE during the TT; MR_TTreq_, required metabolic rate during the TT; MR_ae_peak_, peak aerobic metabolic rate during the TT; ΣO_2_ deficit, accumulated oxygen deficit. The mass used is system mass (i.e., the sum of body mass and skiing equipment mass)*.

*F-values, P-values, and eta squared effect size (η^2^) were obtained by a one-way ANOVA*.

* *Main effect between methods (P < 0.05)*.

$ *Main effect between methods (P < 0.001*).

a *Statistically significantly different from 8+Y_LIN_ (P < 0.05)*.

b *Statistically significantly different from 8-Y_LIN_ (P < 0.05)*.

c *Statistically significantly different from 8+Y_POL_ (P < 0.05)*.

d *Statistically significantly different from 8-Y_POL_ (P < 0.05)*.

e *Statistically significantly different from GE_AVG_ (P < 0.05)*.

f *Statistically significantly different from GE_LAST_ (P < 0.05)*.

### Data of the 4-min Time Trial

During DP (at 1.5°), the participants completed the 4-min time trial at average speed of 25.9 ± 1.2 km·h^−1^ and an average power output of 277 ± 28 W. For DS (at 6.5°), the 4-min time trial speed was 13.6 ± 0.5 km·h^−1^ resulting in an average power output of 406 ± 38 W. During the time trials, the skiers' reached a $\dot{\text{V}}$O_2peak_ of 66 ± 4 ml·kg^−1^·min^−1^ (5.1 ± 0.5 L·min^−1^) at an RER of 1.12 ± 0.07 in DP; and 69 ± 4 ml·kg^−1^·min^−1^ (5.3 ± 0.6 L·min^−1^) at an RER of 1.15 ± 0.05 in DS ($\dot{\text{V}}$O_2peak_: *P* = 0.001, ES_*Hg_av*_ = −0.7; and RER: *P* = 0.072, ES_*Hg_av*_ = −0.6). Peak ventilation rates during the respective time trials in DP and DS were 185 ± 23 and 189 ± 21 L·min^−1^ (*P* = 0.176, ES_*Hg_av*_ = −0.2). Peak heart rates in DP and DS were 182 (IQR = 178–188) and 186 (IQR = 183–191) beats·min^−1^ (*P* < 0.001). The blood lactate concentrations 2-min prior to the time trial were 2.06 ± 0.54 and 1.88 ± 0.78 mmol·L^−1^ for DP and DS, respectively (*P* = 0.408, ES_*Hg_av*_ = 0.3), and 11.72 ± 2.07 and 12.56 ± 2.50 mmol·L^−1^ 2-min after the time trial for DP and DS, respectively (*P* = 0.216, ES_*Hg_av*_ = −0.3). Immediately after the respective time trials, median RPE values were 9 (IQR = 7–10) for DP and 10 (9–10) for DS (*P* = 0.016).

**Figure 6:**
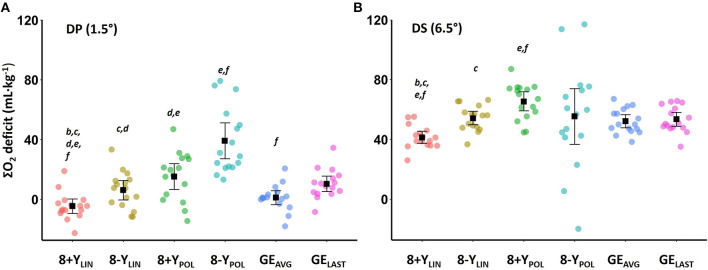
Mean accumulated oxygen (ΣO_2_) deficits and 95% confidence interval together with individual data (colored dots) determined during a 4-min roller-skiing time trial using **(A)** the double poling (DP) sub-technique at 1.5° and **(B)** the diagonal stride (DS) sub-technique at 6.5° using six different methods of calculation. 8+*Y*_LIN_ and 8-Y_LIN_, the 8 × 4-linear methods with the baseline metabolic rate as a Y-intercept either included (8+Y) or excluded (8-Y); 8+Y_POL_ and 8-Y_POL_, the 8 × 4-min polynomial methods with the baseline metabolic rate as a Y-intercept either included (8+Y) or excluded (8-Y); GE_AVG_, the gross efficiency method based on the average of eight submaximal stages; GE_LAST_, the gross efficiency method based on the last submaximal stage. The letters (^***b*−*f***^) indicate statistically significant differences (SSD, *P* < 0.05) between the six methods of calculation: ^***b***^ = SSD from 8-Y_LIN_, ^***c***^ = SSD from 8+Y_POL_, ^***d***^ = SSD from 8-Y_POL_, ^***e***^ = SSD from GE_AVG_, ^***f***^ = SSD from GE_LAST_.

Data from the individual regressions between speed (km·h^−1^) and relative metabolic rate (W·kg^−1^) (8+*Y*_LIN_, 8-Y_LIN_; 8+Y_POL_, 8-Y_POL_), together with the estimated average GE values, estimated metabolic requirements and anaerobic capacities during the time trial of the six different methods are presented in Table 2. The two-way ANOVA (6 × 2) showed main effects between the anaerobic capacity estimates for both computational method and sub-technique (method: F_1, 20_ = 18.59, *P* < 0.001, η^2^ = 0.55; sub-technique: F_1, 15_ = 135.51, *P* < 0.001, η^2^ = 0.90), and an interaction effect between computational method and sub-technique (interaction effect: F_1, 21_ = 15.47, *P* < 0.001, η^2^ = 0.51). All anaerobic capacity estimates (expressed as ΣO_2_ deficits) of the 4-min time trial are presented in Figure 6 for DP (main effect: F_2, 27_ = 42, *P* < 0.001, η^2^ = 0.74) and DS (main effect: F_1, 77_ = 6, *P* = 0.028, η^2^ = 0.27). As shown in Figure 6A, the 8-Y_POL_ method was the only procedure that did not result in any negative ΣO_2_ deficit value for any of the tested skiers during DP.

**Figure 7:**
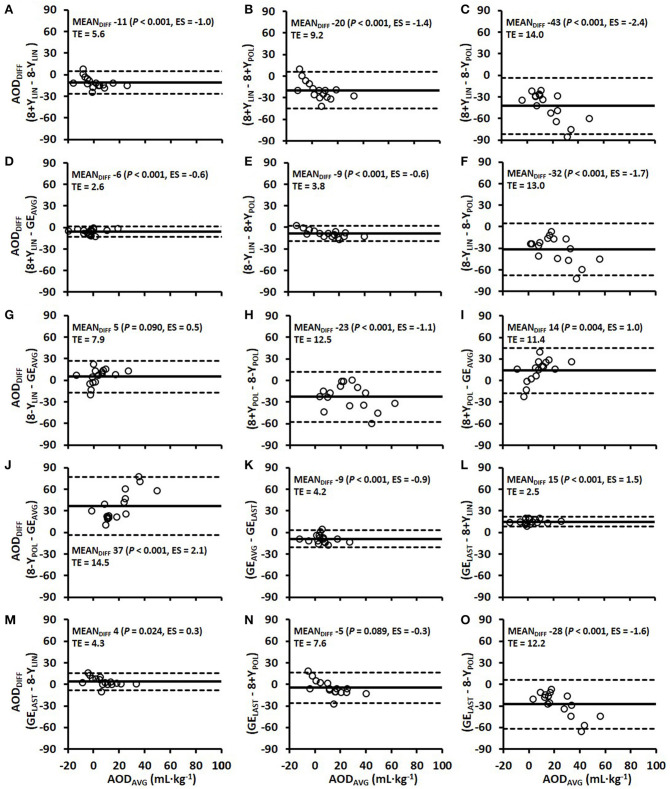
Bland-Altman plots for the six various models of estimating the accumulated oxygen deficit (AOD) associated with the 4-min time trial using the double poling sub-technique **(A–O)**. Bland-Altman plots represent the mean difference (MEAN_DIFF_) in the AOD ± 95% (1.96 SD) limits of agreement between the methods. Abbreviations: AOD_DIFF_, the difference in AOD; TE, typical error; ES, Hedges's *g*_*av*_ effect size, 8+*Y*_LIN_ and 8-Y_LIN_, the 8 × 4-min linear maximal accumulated O_2_ deficit methods with the baseline $\dot{\text{V}}{\text{O}}_{2}$ as a Y-intercept either included (8+Y) or excluded (8-Y); 8+Y_POL_ and 8-Y_POL_, the 8 × 4-min polynomial (second degree) maximal accumulated O2 deficit methods with the baseline $\dot{\text{V}}{\text{O}}_{2}$ as a Y-intercept either included (8+Y) or excluded (8-Y); GE_AVG_, the gross efficiency method based on the average of eight submaximal stages; GE_LAST_, the gross efficiency method based on the last submaximal stage.

**Figure 8:**
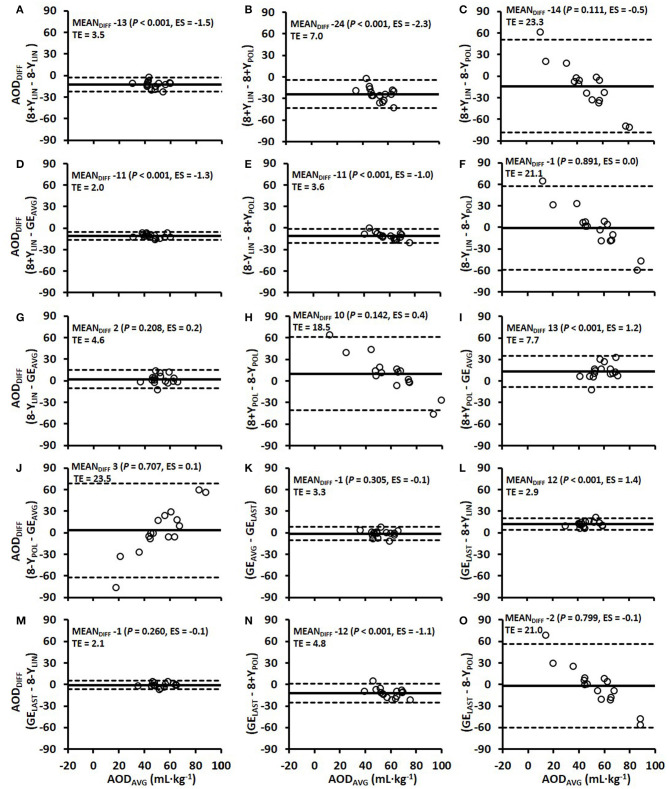
Bland-Altman plots for the six various models of estimating the accumulated oxygen deficit (AOD) associated with the 4-min time trial using the diagonal stride sub-technique **(A–O)**. Bland-Altman plots represent the mean difference (MEAN_DIFF_) in the AOD ± 95% (1.96 SD) limits of agreement between the methods. Abbreviations: AOD_DIFF_, the difference in AOD; TE, typical error; ES, Hedges's *g*_*av*_ effect size, 8+*Y*_LIN_ and 8-Y_LIN_, the 8 × 4-min linear maximal accumulated O_2_ deficit methods with the baseline $\dot{\text{V}}{\text{O}}_{2}$ as a Y-intercept either included (8+Y) or excluded (8-Y); 8+Y_POL_ and 8-Y_POL_, the 8 × 4-min polynomial (second degree) maximal accumulated O2 deficit methods with the baseline $\dot{\text{V}}{\text{O}}_{2}$ as a Y-intercept either included (8+Y) or excluded (8-Y); GE_AVG_, the gross efficiency method based on the average of eight submaximal stages; GE_LAST_, the gross efficiency method based on the last submaximal stage.

**Figure 9:**
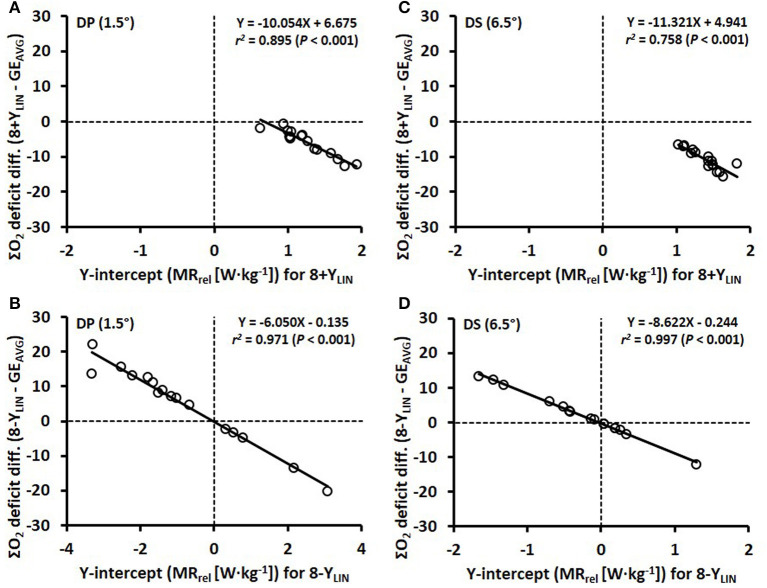
Scatter plots between the Y-intercept values for the 8 × 4-linear methods with the baseline metabolic rate (MR) as a Y-intercept either included (8+Y_LIN_) or excluded (8-Y_LIN_) in the model (*x*-axis) and the accumulated oxygen deficit difference (ΣO_2_ deficit diff.) vs. the gross efficiency method based on the average of eight submaximal stages (GE_AVG_) (*y*-axis). **(A,B)** show the results for double poling (DP) and **(C,D)** show results for diagonal stride (DS).

Comparisons of the anaerobic capacity estimates from the 4-min time trial of the different models (8+*Y*_LIN_, 8-Y_LIN_, 8+Y_POL_, 8-Y_POL_, GE_AVG_ and GE_LAST_) in DP and DS are presented in Figures 7, 8. As shown in Figures 7, 8, 8+*Y*_LIN_ generated, in both DP and DS, clearly lower anaerobic capacities than 8-Y_LIN_ and the linear models resulted in markedly lower anaerobic capacities than the polynomial models for most of the comparisons. Figure 9 shows that the Y-intercept values of the 8+*Y*_LIN_ and 8-Y_LIN_ methods are highly related to the difference in the ΣO_2_ deficit estimates between the 8+*Y*_LIN_ or 8-Y_LIN_ MAOD methods and the GE_AVG_ method, with *r*^2^ values ≥ 0.758.

## Discussion

The current study is the first study that investigated both the effect of computational method and skiing sub-technique on the anaerobic capacity of cross-country skiers. We found that (1) the effect of computational method differed between the DP and DS sub-techniques; (2) the second degree polynomial MAOD method excluding a fixed Y-intercept (8-Y_POL_) described the relationship between speed and submaximal metabolic rate best in DP, while GE_LAST_ showed the closest agreement in anaerobic capacity with 8-Y_POL_ in DP; (3) the linear MAOD method excluding a fixed Y-intercept (8-Y_LIN_) described the relationship between speed and submaximal metabolic rate best in DS and showed the closest agreement with GE_LAST_.

### Different Effect of Computational Method in DP and DS Sub-techniques

Interestingly, the effect of computational method differed between the DP and DS sub-techniques and it seems more complicated to estimate the anaerobic capacity in DP than in DS. This is due to the polynomial relationship between speed and submaximal metabolic rate, which also results in GE to be speed-dependent (i.e., non-linear) (see Figures 2–4). Therefore, none of the more conventional methods for estimating the anaerobic capacity (i.e., the linear MAOD methods or both GE methods) can be recommended for DP, which has also been indicated in previous publications (Sandbakk et al., 2016; Andersson et al., 2017) – but not systematically analyzed. Therefore, when evaluating the anaerobic capacity in DP, the non-linear relationships for speed vs. metabolic rate and the non-linear speed-dependency of GE suggests the use of alternative methods, such as the second-degree polynomial method (i.e., 8-Y_POL_) described in the current study, or, alternatively, the method described by Beneke et al. (2002).

In DS, the 8-Y_LIN_, GE_AVG_ and GE_LAST_ methods resulted in very similar average values of the ΣO_2_ deficit, due to the linear relationship between speed and metabolic rate, as well as the speed independent GE (see Figures 2–4). Although mean ΣO_2_ deficit values were similar for the 8-Y_LIN_, GE_AVG_ and GE_LAST_ in DS, agreements between all the different methods in DP and DS were generally low with rather high mean differences and typical errors. This suggests that the different methods should not be used interchangeably, which is in line with previous findings (Noordhof et al., 2011; Andersson and McGawley, 2018).

### Evaluating the Accuracy of the Different Methods

Due to the lack of a gold standard procedure for evaluating the accuracy of the different methods for estimating the anaerobic capacity, the precision of the different methods was based on the fit of the regression line (i.e., SEE values). In addition, the root mean square error for the GE_REG_, i.e., estimated GE based on the regression equation, compared to the actually measured GE was used (see Figure 5). In DP, the 8-Y_POL_ method generated the lowest SEE based on the regression equation as well as the lowest root mean square error for the GE_REG_ vs. the measured GE. However, for DS, the 8-Y_POL_ method generated an unreasonably high between-athlete variation of the anaerobic capacity (see Figures 4H, 6B), which to some extent also highlights a limitation of using polynomial methods for estimating anaerobic capacity. This might be related to the fact that a polynomial regression follows the data-points more closely than in a linear regression. The latter methodological problem is likely one explanation for the high between-athlete variation in the ΣO_2_ deficit estimate for the 8-Y_POL_ method in DP (see Figure 6A). Therefore, when estimating a supramaximal metabolic demand based on extrapolation of a polynomial relationship between speed and MR, it is perhaps wise to add more submaximal stages at the upper part of the submaximal protocol, or using the methodological concept described by Beneke et al. (2002). Even though the current study was not designed for estimating anaerobic capacity as previously described by Beneke et al. (2002) and Guidetti et al. (2007), a simplified calculation resulted in data (unreported) that were relatively similar to the average values obtained with the 8-Y_POL_ method in DP and all the methods in DS. By assuming that a 1 mmol·L^−1^ delta increase in blood lactate concentration during the 4-min time trial (pre vs. post measures) is equivalent to an ΣO_2_ deficit of 3 mL·kg^−1^ (di Prampero, 1981) and that the lactic anaerobic contribution to the total ΣO_2_ deficit is 67% (Medbø et al., 1988); ΣO_2_ deficits were calculated to 43 ± 9 and 48 ± 12 mL·kg^−1^ in DP and DS, respectively. Therefore, due to the problems with non-linearity for the submaximal speed-$\dot{\text{V}}{\text{O}}_{2}$ relationship in DP, the alternative method suggested by Beneke et al. (2002) using measurements of blood lactate concentration and the fast component of the post-exercise $\dot{\text{V}}{\text{O}}_{2}$-recovery could be a useful method for estimating the ΣO_2_ deficit during supramaximal DP exercise.

Including a forced Y-intercept using either $\dot{\text{V}}{\text{O}}_{2}$ measured at baseline or an arbitrary value has previously been applied in the MAOD method, which has been suggested to increase the precision of the estimated $\dot{\text{V}}{\text{O}}_{2}$ demand (Medbø et al., 1988; Russell et al., 2000, 2002; Bickham et al., 2002). However, as previously addressed by Bangsbo (1992), one could argue why a resting value of $\dot{\text{V}}{\text{O}}_{2}$ should be perfectly aligned with the speed-$\dot{\text{V}}{\text{O}}_{2}$ relationship during submaximal exercise. In the current study, the inclusion of a Y-intercept value in the linear regression between speed and metabolic rate resulted in significantly lower metabolic demands during the time trial for both sub-techniques, which also confirms previous observations by Andersson and McGawley (2018). As shown in Table 2, the SEEs, Y-intercepts and slopes were significantly different for the 8+*Y*_LIN_ than the 8-Y_LIN_ in both DP and DS. The inclusion of a Y-intercept value of metabolic rate resulted in considerably lower slopes of the regression lines in DP and DS with lower delta efficiencies of 2.6 and 2.4 percentage points, respectively. As shown in Figures 5A,C, the GE_REG_ for the 8+*Y*_LIN_ method showed relationships where GE increased linearly with increasing speed for both DP and DS and the root mean square errors for the GE_REG_ vs. GE were significantly higher for the 8+*Y*_LIN_ than the 8-Y_LIN_. Altogether, these results indicate that when including a Y-intercept value for baseline metabolic rate (i.e., at rest) in the linear regression between speed and submaximal metabolic rate, the estimated supramaximal metabolic requirement is likely to be underestimated for DP and DS roller-skiing on a treadmill. Therefore, in order to create a robust relationship between speed and submaximal metabolic rate, it is probably wise to include several submaximal stages than simply adding a Y-intercept, or alternatively for DS using one of the GE methods, preferably the GE_LAST_ method.

As opposed to the linear and polynomial MAOD methods, the GE_AVG_ and GE_LAST_ methods assume that the GE values during the submaximal exercise bouts remain relatively constant, i.e., a low variation between stages and no upward or downward trends. Due to the non-linear relationship between speed and GE in DP (see Figure 2B), it is likely that none of the GE methods would result in valid and reliable estimates of anaerobic capacity and the use of this method in DP should, therefore, be discouraged. In the current study, an interesting unreported result was the unrealistically high anaerobic capacity values (expressed as an ΣO_2_ deficit) of 83 ± 86 mL·kg^−1^, also with vast between-athlete variation, obtained for DP when using a polynomial speed-GE regression equation method for instantaneous extrapolation of GE up to the time trial speed. In theory, such a method would be relatively similar to the 8-Y_POL_ method. However, the unrealistic values obtained with such a method was probably partly related to the substantially higher SEE values for a speed-GE relationship based on a polynomial regression and should therefore not be recommended.

It is logical that if GE would be independent of speed, GE_AVG_ and GE_LAST_ methods would yield exactly similar anaerobic capacity values and that the root mean square errors for the GE_AVG_ and GE_LAST_ vs. the GE values at all the separate submaximal stages would be zero. Although GE remained relatively constant, at the group level, across all submaximal stages in DS (see Figure 2B), small within-athlete variations in GE between the stages resulted in a disagreement between the GE_AVG_ and GE_LAST_ methods, with a typical error in the ΣO_2_ deficit of 3.3 mL·kg^−1^ (see Figure 8K). Interestingly, for the comparison of the ΣO_2_ deficit estimates in DS, the GE_LAST_ method showed a relatively good agreement with the 8-Y_LIN_ method, as the average values were similar and the typical error was relatively low, at 2.1 mL O_2_eq·kg^−1^ (Figure 8M). Due to the higher agreement between the GE_LAST_ and 8-Y_LIN_ than between the GE_AVG_ and 8-Y_LIN_ in DS, it might be possible that the GE_LAST_ method is more adequate than the GE_AVG_ method for DS cross-country skiing. In addition, the GE_LAST_ method is more time-efficient to use, as it only requires one submaximal stage performed at a relatively high submaximal exercise intensity and is, hence, analogous to the GE concept used for estimating anaerobic capacity during cycle ergometry (Noordhof et al., 2011).

### The Difference in Anaerobic Capacity Estimated With the MAOD and GE Methods

A novel finding of the current study is the results presented in Figure 9 showing that the value of the Y-intercept is linearly related to the mean difference in the ΣO_2_ deficits between the 8+*Y*_LIN_ and 8-Y_LIN_ methods vs. the GE_AVG_ method. Hence, the mean differences and typical errors presented in Figures 7, 8 for the 8+*Y*_LIN_ and 8-Y_LIN_ vs. the GE_AVG_ and GE_LAST_ methods can mainly be explained by the average Y-intercept values and the between-athlete variability in Y-intercept values for the linear MAOD methods. Therefore, a smaller range in Y-intercept values results in a lower typical error, while an average Y-intercept value deviating from zero would result in a systematic mean difference for the linear MAOD methods vs. the GE_AVG_ or GE_LAST_ method. Moreover, if the Y-intercept value of the linear regression equation between submaximal power output and metabolic rate was zero, the delta efficiency and the GE values for the submaximal stages as based on the regression equation would be exactly similar. However, if the Y-intercept of a MAOD regression is positive, GE values derived from the regression would increase with higher exercise intensities, while the contrary would be observed for a negative Y-intercept (i.e., a decreasing GE with increasing speed). Hence, the MAOD method does not assume a constant GE; that is only the case if the Y-intercept value of the MAOD regression is zero, a finding that adds to the understanding of the disagreements between the different MAOD and GE methods.

For cycle ergometry, GE usually increases with increasing power output during low- to moderate-intensity submaximal exercise, due to the gradually diminishing relative effect of baseline energy metabolism (i.e., metabolic rate at rest) on the total energy metabolism (Ettema and Lorås, 2009), but has been shown to plateau at a relatively high submaximal power output (de Koning et al., 2012). However, in the current study, GE was found to be relatively constant with increasing power output during DS, while during DP an inverted U-shape relationship was observed between power output and GE. Therefore, also the exercise mode should be considered when assuming potential changes in GE with increasing power output. Moreover, recent studies on well-trained cyclists have shown that GE declines during supramaximal cycle ergometry (de Koning et al., 2013; Noordhof et al., 2015), if this also applies to the both sub-techniques studied in the current study, remains to be evaluated in future studies. However, based on the GE data presented in Figure 2 for DP and DS, it is unlikely that GE would have increased during the supramaximal time trial for both DP and DS.

### Methodological Discussion

Different intensities, durations and number of submaximal exercise bouts have been used when constructing the linear regression line needed for the MAOD method (Green and Dawson, 1993, 1996; Noordhof et al., 2010). In the current study, a continuous submaximal protocol consisting of eight 4-min stages with exercise intensities ranging between ~47–78% of $\dot{\text{V}}$O_2peak_ was used (for details, see Table 1). One difference between cross-country skiing and other exercise modes is the different sub-techniques that are used for different speed and incline combinations, which to some extent also narrows the intensity range for the submaximal stages within each sub-technique. For instance, in DS, a speed slower than the speed at the first submaximal stage in the current study was considered to change the technical execution of the sub-technique too much, which resulted in a lowest exercise intensity of 49% of $\dot{\text{V}}$O_2peak_. On the other hand, higher exercise intensities than ~80% of $\dot{\text{V}}$O_2peak_ are problematic due to the gradually increasing anaerobic energy contribution leading to an underestimated metabolic requirement (Green and Dawson, 1993; Noordhof et al., 2010). There is always uncertainty related to the extrapolation of a submaximal speed-metabolic rate relationship to supramaximal exercise intensities. This problem is likely to be larger for DP than DS due to the non-linear speed-metabolic rate relationship during DP (see Figures 3, 4). In the current study, the submaximal exercise intensities relative to $\dot{\text{V}}$O_2peak_ could have been more exactly targeted on an individual basis. This by adding a pretest for evaluating the physiological response in each sub-technique, thus enabling a maximized submaximal exercise intensity spectrum with the potential advantage of a more accurate estimate of the supramaximal metabolic requirement and values of anaerobic capacity.

In the literature, both continuous and discontinuous submaximal protocols have been used for estimating the ΣO_2_ deficit when using the MAOD method (Medbø et al., 1988; Green and Dawson, 1993; Noordhof et al., 2010). A continuous protocol is more time-efficient and practical but may be problematic due to the potential of a gradually increasing $\dot{\text{V}}{\text{O}}_{2}$ slow component during exercise. It is proposed that the slow component of $\dot{\text{V}}{\text{O}}_{2}$ is in part related to a progressive loss in muscle efficiency at intensities above the lactate threshold (Jones et al., 2011). Although the current study was not designed for evaluating the magnitude of the slow component during DP and DS submaximal roller-skiing, the average $\dot{\text{V}}{\text{O}}_{2}$ uptakes at min 3 and 4 of each separate submaximal stage were very similar (on average 0.5 ± 0.3 and 0.0 ± 0.3% higher at the fourth than the third minute in DP and DS, respectively). In addition, there were no tendencies of larger differences at the highest submaximal intensities, which would indicate a relatively negligible slow component. In a previous study by Björklund et al. (2011), both elite- and moderately-trained cross-country skiers showed no drift in $\dot{\text{V}}{\text{O}}_{2}$ while completing a continuous variable-intensity test comprising of 5-6 bouts with 3-min high-intensity exercise (90% of $\dot{\text{V}}$O_2max_), each interspersed with 6 min of exercise at 70% of $\dot{\text{V}}$O_2max_ using the DS sub-technique. The slow component of $\dot{\text{V}}{\text{O}}_{2}$ is partly explained by a loss in muscular efficiency (Jones et al., 2011), therefore, it is likely to assume that a 4-min supramaximal exercise would result in a declining GE similar to that observed for cycle ergometry exercise (de Koning et al., 2013; Noordhof et al., 2015). However, unpublished data from our laboratory on well-trained cross-country skiers showed no differences in GE before and after a 3-min uphill DS time trial. All these data suggest that the magnitude of any developing slow component would probably be rather small for DP and DS treadmill roller-skiing. Since the magnitude of the $\dot{\text{V}}{\text{O}}_{2}$ slow component is also related to the exercise mode (Billat et al., 1998; Jones et al., 2011), the $\dot{\text{V}}{\text{O}}_{2}$ slow component response for different sub-techniques of cross-country skiing needs further evaluation in future studies.

When deciding the choice of method used for estimating the anaerobic capacity both the exercise mode (and sub-technique used in cross-country skiing) and the fitness level of the participants should be considered. The gradually decreasing relative impact of baseline metabolism on GE with increasing exercise intensity (or power output) is one factor explaining the curvature of the power output-GE relationship (Ettema and Lorås, 2009). Therefore, in a participant group of recreationally active people with relatively low aerobic fitness, it is likely to assume that GE would increase with increasing submaximal power output and hence, the GE method would likely be insufficient. In addition, a low fitness level of the participants results in a low range of submaximal exercise intensities, which might limit the accuracy of the MAOD method. Finally, as based on the goodness of fit of the linear regression lines in the MAOD method and the speed-independency of GE, we can choose a particular method to calculate the anaerobic capacity. However, we remain uncertain if the estimated anaerobic capacity reflects the real anaerobic capacity, as neither of the methods has been validated during whole-body exercise, due to the lack of a gold-standard procedure for validation (Noordhof et al., 2010).

## Perspectives and Conclusions

The current study provides new insight in which methodological concepts could be used for determining anaerobic capacity in the two most important sub-techniques of classic cross-country skiing. Our data indicate that different methodological concepts should be used to estimate the anaerobic capacity in DP and DS. In DP, a polynomial MAOD method seems to be the preferred method for estimating the ΣO_2_ deficit, whereas the 8-Y_LIN_, GE_AVG_, and GE_LAST_ can all be used for DS, with GE_LAST_ being the least time-consuming method, as it only requires the completion of one submaximal exercise bout and a supramaximal exercise test. However, due to the relatively high disagreements between methods, different methods should not be used interchangeably when testing athletes on a regular basis. The traditional view that a baseline value for resting metabolism (i.e., Y-intercept) should be included in the MAOD regression can, as based on the results presented in the current study for the DP and DS sub-techniques, be discarded.

## Data Availability Statement

The datasets generated for this study are available on request to the corresponding author.

## Ethics Statement

The studies involving human participants were reviewed and approved by The Regional Ethical Review Board of Umeå University, Umeå, Sweden (#2018-154-31 M). The patients/participants provided their written informed consent to participate in this study.

## Author Contributions

EA designed the study. EA and NL collected the data and performed the statistical analysis. EA, DN, and NL interpreted the results, wrote the first draft, revised the manuscript, approved the final version to be published, and agreed to be accountable for all aspects of the work.

### Conflict of Interest

The authors declare that the research was conducted in the absence of any commercial or financial relationships that could be construed as a potential conflict of interest.
